# Acute Encephalopathy Due to Polypharmacy Interactions With the Use of Paxlovid: A Case Report

**DOI:** 10.7759/cureus.36535

**Published:** 2023-03-22

**Authors:** Samira Khan, Joseph Fama

**Affiliations:** 1 Behavioral Health, West Viriginia University (WVU) Berkeley Medical Center, Martinsburg, USA; 2 Pharmacotherapy, West Virginia University (WVU), Martinsburg, USA

**Keywords:** medication interaction, geriatric, medical, psychiatry, covid-19

## Abstract

Paxlovid is an oral therapy in the treatment of COVID-19. It has been authorized by the Food and Drug Administration (FDA) to be used under the Emergency Use Authorization Act (EUAA) for the treatment of mild-to-moderate COVID-19 in adults and pediatric patients (12 years of age and older weighing at least 40 kg) who are at high-risk for progression to severe COVID-19 and remains under extended review for New Drug Application (NDA). Paxlovid comprises two anti-viral medications: nirmatrelvir and ritonavir, which cause significant drug-drug interactions. In this case report, an elderly patient received Paxlovid and had medication interactions with benzodiazepines and narcotics leading to altered mental status.

## Introduction

Since the beginning of the COVID-19 pandemic, many options to either prevent or treat the disease have been proposed and tried. Paxlovid represents a pharmaceutical breakthrough as it is the first oral therapy for the treatment of SARS-CoV-2. Being utilized under the Emergency Use Authorization Act (EUAA), Paxlovid is not approved by FDA. Nevertheless, it is the most recently developed medication for the treatment of mild to moderate COVID-19 infections among patients at risk of progressing to serious disease. Furthermore, it can be used in patients aged 12 years and older who weigh over 40 kg [1]. In our report, we focus on several important drug-drug interactions with Paxlovid.

## Case presentation

The patient is a 74-year-old female with a past medical history of hypertension and chronic obstructive pulmonary disease (COPD), is wheelchair-bound, and had denied a significant past psychiatric history. She was recently treated for *Clostridioides difficile*, UTI, and COVID-19 symptoms at an outside facility. The patient was treated for COVID-19 with Paxlovid and discharged home with antibiotics for *C. diff *and UTI. The patient was admitted to our facility within 24 hours of that discharge due to concerns of weakness and an altered mental status (AMS). The medical team had ruled out medical causes for her chief complaints and consulted psychiatry for the AMS. A preliminary chart review was conducted prior to assessing the patient in the medical unit. Her home medications consisted of diazepam, acetaminophen-codeine, tramadol, gabapentin, pravastatin, nystatin, albuterol sulfate, bisoprolol fumarate, cholecalciferol, fluticasone propionate-salmeterol, ipratropium-albuterol, magnesium oxide, tiotropium bromide, and triamterene hydrochlorothiazide. The patient was not on antibiotics at the time of her ER presentation. The patient’s liver enzymes were significantly elevated as well, so the primary team was advised of the polypharmacological interactions and advised to either hold or discontinue medications that may be impacting the liver enzymes. The primary team held benzodiazepine, tramadol, codeine, and statins. The patient’s mental status started to return to baseline within 24 hours of holding the medications. She was soon discharged home at her baseline.

## Discussion

As previously mentioned, Paxlovid is comprised of two anti-viral protease inhibitor medications: nirmatrelvir and ritonavir. Nirmatrelvir has activity against SARS-CoV-2 main protease, which leads to decreased viral replication. Ritonavir, on the other hand, has no activity against COVID-19 but is instead used because it is a potent cytochrome P450 3A (CYP3A) inhibitor, which leads to increased levels of nirmatrelvir and delays its clearance [2,3]. Ritonavir is used as a pharmacokinetic enhancer or boosting agent and is required in this combination for nirmatrelvir to be able to reach its therapeutic levels to treat COVID-19 [3]. It should be noted that medications in this class are well-known for many significant drug-drug interactions.

The patient’s age, duplicate narcotic medications, and her taking more than the recommended amounts of prescription benzodiazepine put her at high risk for negative health outcomes. Both of Paxlovid’s components have a known drug interaction to increase levels of diazepam through CYP3A4 inhibition as well as increasing tramadol serum concentration levels. Codeine may also have its levels altered by Paxlovid [3]. These interactions were most likely the primary cause of the patient’s onset of confusion.

The National Health Institute (NIH) COVID treatment guidelines feature an extensive list of drug-drug interactions with Paxlovid (see Appendices) [4]. In addition, as of August 2022, the FDA released an updated checklist for potential risk factors and drug-drug interactions to be reviewed before administrating Paxlovid to patients (Table 1) [5]. Although many medications can be safely taken while on Paxlovid, the drug-drug interaction list should still be referred to prior to and during treatment [4].

**Table 1:** List of clinically significant drug interactions including contraindicated drugs for Paxlovid XXX: Coadministration of this drug with Paxlovid is contraindicated. For further information, refer to the Fact Sheet for Healthcare Providers and the individual Prescribing Information for the drug [6]. *** Coadministration of this drug with Paxlovid should be avoided and/or holding of this drug,  dose adjustment of this drug,  or special monitoring is necessary.  Consultation with the prescriber of the potentially interacting drug is recommended. For further information, refer to the Health Care Provider Fact  Sheet and the Individual Prescribing Information for the drug [6].

Drug	Drug Class	Interaction Code
abemaciclib	Anticancer drug	***
alfuzosin	Alpha 1-adrenoreceptor antagonist	XXX
aliskiren	Cardiovascular agent	***
amiodarone	Antiarrhythmic	XXX
amlodipine	Calcium channel blocker	***
apalutamide	Anticancer drug	XXX
apixaban	Anticoagulant	***
aripiprazole	Neuropsychiatric agent	***
avanafil	PDE5 inhibitor	***
bedaquiline	Antimycobacterial	***
betamethasone	Systemic corticosteroid	***
brexpiprazole	Neuropsychiatric agent	***
bosentan	Endothelin receptor antagonist	***
budesonide	Systemic corticosteroid	***
bupropion	Antidepressant	***

## Conclusions

The introduction of Paxlovid as an oral therapy represents a significant step forward in the treatment of COVID-19. The main advantage of oral therapies over treatments completed in infusion centers allows the patient to save time and money. However, as it is a new medication with limited research and data, it will be the medical teams’ responsibility to diligently monitor for significant drug-drug interactions when prescribing and administering Paxlovid and allow room for modifications as they see fit.

## Appendices

**Table 2:** Select outpatient medications that have clinically relevant drug-drug interactions with ritonavir-boosted nirmatrelvir (Paxlovid)

Prescribe Alternative COVID-19 Therapy			
For these medications, management strategies are not possible or feasible, or the risks outweigh the potential benefits.			
Anticonvulsants Carbamazepine Phenobarbital Phenytoin Primidone Anti-infectives Glecaprevir/pibrentasvir Rifampin Rifapentine	Immunosuppressants Voclosporin Cardiovascular Amiodar Temporarily Withhold Concomitant Medication, if Clinically Appropriate Withhold these medications during ritonavir-boosted nirmatrelvir treatment and for at least 2–3 days after treatment completion. They may need to be withheld for longer if the patient is an adult of advanced age or the medication has a long half-life. If withholding is not clinically appropriate, use an alternative concomitant medication or COVID-19 therapy. Anticoagulants Rivaroxaban^d ^Anti-infectives Erythromycin BPH Alfuzosin Silodosin Cardiovascular Aliskiren Ranolazine Ticagrelor^b^ Vorapaxar Immunosuppressants^e^ Everolimus Sirolimus TacrolimusLipid-modifiers Atorvastatin^f^ Lomitapide Lovastatin^f^ Rosuvastatin^f^ Simvastatin^f ^Migraine Eletriptan Rimegepant Ubrogepant Neuropsychiatric Suvorexant Triazolam^g^ Erectile dysfunction AvanafilRespiratory Salmeterol Miscellaneous Certain chemotherapeutic agents^c^ Colchicine^h^ Finerenone Flibanserin Naloxegol Adjust Concomitant Medication Dose and Monitor for Adverse Effects Consult the Liverpool COVID-19 Drug Interactions website or the University of Waterloo/University of Toronto drug interaction guide for specific dosing recommendations.^i^ If the dose of the concomitant medication cannot be adjusted, withhold the medication (if clinically appropriate) or use an alternative concomitant medication or COVID-19 therapy. Anticoagulants Apixaban Dabigatran Edoxaban Anti-infectives Clarithromycin Itraconazole Ketoconazole Maraviroc Rifabutin BPH TamsulosinCardiovascular Cilostazol Digoxin Mexiletine Diabetes Saxagliptin Erectile dysfunction Sildenafil Tadalafil Vardenafil Immunosuppressants Cyclosporine^e^ Dexamethasone^j^ Fedratinib Ruxolitinib Tofacitinib Upadacitinib Migraine Almotriptan^h ^Neuropsychiatric Alprazolam^g^ Aripiprazole Brexpiprazole Buspirone Cariprazine Chlordiazepoxide^g^ Clobazam^g^ Clonazepam^g^ Clorazepate^g^ Neuropsychiatric, continued Diazepam^g^ Estazolam^g^ Flurazepam^g^ Iloperidone Lumateperone Pimavanserin Quetiapine TrazodonePain Fentanyl Hydrocodone Oxycodone Pulmonary hypertension Riociguat Miscellaneous Certain chemotherapeutic agents^c^ Darifenacin Elexacaftor/tezacaftor/ivacaftor Eluxadoline Ivacaftor Solifenacin Tezacaftor/ivacaftor one Clopidogrel^a,b^ Disopyramide Dofetilide Dronedarone Eplerenone Flecainide	Cardiovascular, continued Ivabradine Propafenone Quinidine Neuropsychiatric Clozapine Lurasidone Midazolam (oral) Pimozide	Pulmonary hypertension Sildenafil Tadalafil Vardenafil Miscellaneous Bosentan Certain chemotherapeutic agents^c^ Ergot derivatives Lumacaftor/ivacaftor St. John’s wort Tolvaptan

Continue Concomitant Medication and Monitor for Adverse Effects			
Pre-emptive dose adjustment is not required but may be considered based on an individualized assessment of the patient’s risk for adverse reactions. Educate patients about potential adverse effects. Consult the Liverpool COVID-19 Drug Interactions website or the University of Waterloo/University of Toronto drug interaction guide for monitoring guidance and dose adjustment information as needed.^i^			
Anticoagulants WarfarinAnti-infectives Brincidofovir^k^ Cobicistat- or ritonavir-boosted antiretrovirals Isavuconazole Posaconazole VoriconazoleBPH Doxazosin Terazosin	Diabetes Glyburide Cardiovascular Amlodipine Diltiazem Felodipine Nifedipine Sacubitril Valsartan VerapamilMigraine Zolmitriptan	Neuropsychiatric Haloperidol Hydroxyzine Mirtazapine Risperidone Ziprasidone Zolpidem Pain Buprenorphine Hydromorphone Methadone Morphine Tramadol	Miscellaneous Certain chemotherapeutic agents^c^ Certain conjugated monoclonal antibodies^l^ Oxybutynin
Reduced effectiveness of clopidogrel is likely. It may be acceptable to continue clopidogrel if the benefits of using ritonavir-boosted nirmatrelvir outweigh the risk of reduced clopidogrel effectiveness. For patients at very high risk of thrombosis (e.g., those who received a coronary stent within the past 6 weeks), consider prescribing an alternative antiplatelet (e.g., prasugrel, if clinically appropriate) or an alternative COVID-19 therapy. Ritonavir-boosted nirmatrelvir may increase concentrations of some chemotherapeutic agents, leading to an increased potential for drug toxicities. Some chemotherapeutic agents may decrease the effectiveness of ritonavir-boosted nirmatrelvir. Please refer to the FDA EUA fact sheet for ritonavir-boosted nirmatrelvir and the prescribing information for the chemotherapeutic agent and consult the patient’s specialist provider. The University Health Network/Kingston Health Sciences Centre is an additional resource for evaluating drug-drug interactions for chemotherapeutic agents. For patients who are at high risk of arterial or venous thrombosis (e.g., those who had a stroke within the past 3 months with a CHA2DS2-VASc score of 7–9 or a pulmonary embolism within the past month), consult the primary or specialty provider and consider using an alternative anticoagulant such as LMWH or an alternative COVID-19 therapy. For patients with a lower risk for arterial or venous thrombosis, clinicians may consider administering low-dose aspirin while rivaroxaban is being withheld. The use of another COVID-19 therapy may need to be considered. These immunosuppressants have significant drug-drug interaction potential with ritonavir, and they should not be used if close monitoring, including therapeutic drug monitoring, is not feasible. Consult a patient’s specialist providers before coadministering these immunosuppressants and ritonavir-boosted nirmatrelvir. See the American Society of Transplantation statement for more information. Withhold lovastatin and simvastatin for at least 12 hours before initiating ritonavir-boosted nirmatrelvir, during treatment, and for 5 days after treatment completion. Withhold atorvastatin and rosuvastatin at the beginning of treatment with ritonavir-boosted nirmatrelvir and resume after completion of the 5-day course. If withholding a statin is not clinically appropriate (e.g., the patient had a recent myocardial infarction), the doses of atorvastatin and rosuvastatin can be adjusted and continued, and lovastatin and simvastatin should be switched to an alternative statin. The guidance on managing drug-drug interactions between certain benzodiazepines and ritonavir-boosted nirmatrelvir can vary significantly between resources. The guidance in this table is based on the FDA EUA fact sheet for ritonavirboosted nirmatrelvir. Note that abrupt discontinuation or rapid dose reduction of benzodiazepines may precipitate an acute withdrawal reaction.^2^ The risk is greatest for patients who have been using high doses of benzodiazepines over an extended period. ^h^ For patients with hepatic or renal impairment, do not coadminister this medication with ritonavir-boosted nirmatrelvir. ^i^ For medications that are not included on the Liverpool COVID-19 Drug Interactions website or the University of Waterloo/ University of Toronto drug interaction guide, refer to the FDA labels for information on coadministering these medications with ritonavir or other strong CYP3A4 and/or P-gp inhibitors.			
Continue Concomitant Medication and Monitor for Adverse Effects, continued			
^j^ Dexamethasone exposure is expected to increase 2.60-fold when dexamethasone is coadministered with ritonavirboosted nirmatrelvir.^3^ Clinicians should weigh the risks and benefits of continuing the patient’s normal dose of dexamethasone (while monitoring for AEs) versus decreasing the dose. Patients who are receiving higher doses of dexamethasone will be at a greater risk of AEs. ^k^ Patients should take ritonavir-boosted nirmatrelvir at least 3 hours after receipt of brincidofovir. ^l^ Ritonavir-boosted nirmatrelvir interacts with certain conjugated monoclonal antibodies, such as those conjugated to the drug monomethyl auristatin E (or vedotin). These include brentuximab vedotin, enfortumab vedotin, polatuzumab vedotin, and tisotumab vedotin. Before coadministering ritonavir-boosted nirmatrelvir and any of these conjugated monoclonal antibodies, refer to the drug’s FDA prescribing information and consult with the patient’s specialist providers as needed. Key: AE = adverse effect; BPH = benign prostatic hyperplasia; CHA2DS2-VASc = congestive heart failure, hypertension, age, diabetes, stroke, vascular disease; CYP = cytochrome P450; EUA = Emergency Use Authorization; FDA = Food and Drug Administration; LMWH = low-molecular-weight heparin; P-gp = P-glycoprotein			
